# Global fine-resolution data on springtail abundance and community structure

**DOI:** 10.1038/s41597-023-02784-x

**Published:** 2024-01-03

**Authors:** Anton M. Potapov, Ting-Wen Chen, Anastasia V. Striuchkova, Juha M. Alatalo, Douglas Alexandre, Javier Arbea, Thomas Ashton, Frank Ashwood, Anatoly B. Babenko, Ipsa Bandyopadhyaya, Carolina Riviera Duarte Maluche Baretta, Dilmar Baretta, Andrew D. Barnes, Bruno C. Bellini, Mohamed Bendjaballah, Matty P. Berg, Verónica Bernava, Stef Bokhorst, Anna I. Bokova, Thomas Bolger, Mathieu Bouchard, Roniere A. Brito, Damayanti Buchori, Gabriela Castaño-Meneses, Matthieu Chauvat, Mathilde Chomel, Yasuko Chow, Steven L. Chown, Aimee T. Classen, Jérôme Cortet, Peter Čuchta, Ana Manuela de la Pedrosa, Estevam C. A. De Lima, Louis E. Deharveng, Enrique Doblas Miranda, Jochen Drescher, Nico Eisenhauer, Jacintha Ellers, Olga Ferlian, Susana S. D. Ferreira, Aila S. Ferreira, Cristina Fiera, Juliane Filser, Oscar Franken, Saori Fujii, Essivi Gagnon Koudji, Meixiang Gao, Benoit Gendreau-Berthiaume, Charles Gers, Michelle Greve, Salah Hamra-Kroua, I. Tanya Handa, Motohiro Hasegawa, Charlène Heiniger, Takuo Hishi, Martin Holmstrup, Pablo Homet, Toke T. Høye, Mari Ivask, Bob Jacques, Charlene Janion-Scheepers, Malte Jochum, Sophie Joimel, Bruna Claudia S. Jorge, Edite Juceviča, Esther M. Kapinga, Ľubomír Kováč, Eveline J. Krab, Paul Henning Krogh, Annely Kuu, Natalya Kuznetsova, Weng Ngai Lam, Dunmei Lin, Zoë Lindo, Amy W. P. Liu, Jing-Zhong Lu, María José Luciáñez, Michael T. Marx, Amanda Mawan, Matthew A. McCary, Maria A. Minor, Grace I. Mitchell, David Moreno, Taizo Nakamori, Ilaria Negri, Uffe N. Nielsen, Raúl Ochoa-Hueso, Luís Carlos I. Oliveira Filho, José G. Palacios-Vargas, Melanie M. Pollierer, Jean-François Ponge, Mikhail B. Potapov, Pascal Querner, Bibishan Rai, Natália Raschmanová, Muhammad Imtiaz Rashid, Laura J. Raymond-Léonard, Aline S. Reis, Giles M. Ross, Laurent Rousseau, David J. Russell, Ruslan A. Saifutdinov, Sandrine Salmon, Mathieu Santonja, Anna K. Saraeva, Emma J. Sayer, Nicole Scheunemann, Cornelia Scholz, Julia Seeber, Peter Shaw, Yulia B. Shveenkova, Eleanor M. Slade, Sophya Stebaeva, Maria Sterzynska, Xin Sun, Winda Ika Susanti, Anastasia A. Taskaeva, Li Si Tay, Madhav P. Thakur, Anne M Treasure, Maria Tsiafouli, Mthokozisi N. Twala, Alexei V. Uvarov, Lisa A. Venier, Lina A. Widenfalk, Rahayu Widyastuti, Bruna Winck, Daniel Winkler, Donghui Wu, Zhijing Xie, Rui Yin, Robson A. Zampaulo, Douglas Zeppelini, Bing Zhang, Abdelmalek Zoughailech, Oliver Ashford, Osmar Klauberg-Filho, Stefan Scheu

**Affiliations:** 1 German Centre for Integrative Biodiversity Research (iDiv) Halle-Jena-Leipzig, Puschstrasse 4, 04103 Leipzig, Germany; 2 Institute of Biology, Leipzig University, Puschstrasse 4, 04103 Leipzig, Germany; 3 Department of Animal Ecology, Johann Friedrich Blumenbach Institute of Zoology and Anthropology, University of Göttingen, Göttingen, 37073 Germany; 4 Department of zoology and ecology, Institute of Biology and Chemistry, Moscow Pedagogical State University, Kibalchicha 6 B.3, Moscow, 129164 Russia; 5 Environmental Science Center, Qatar University, Doha, Qatar; 6 Department of Soil Science, Centre for Agriculture and Veterinary Science, Santa Catarina State University (UDESC-Lages), Lages, SC Brazil; 7 CEPA Camargo, c/ Ria de Solia 3, ch. 39, 39610 Astillero, Spain; 8 Forest Research, Northern Research Station, Roslin, Midlothian, Scotland EH25 9SY United Kingdom; 9 A.N. Severtsov Institute of Ecology and Evolution, Russian Academy of Sciences, Leninskij prospekt 33, 119071 Moscow, Russia; 10 Patha Bhavan, Visva Bharati,Santiniketan, Birbhum, West Bengal India; 11 Department Animal Science, University of Santa Catarina (UDESC), Chapeco, SC 89815-000 Brazil; 12 Te Aka Mātuatua - School of Science, University of Waikato, Private Bag 3105, Hamilton, 3204 New Zealand; 13 Department of Botany and Zoology, Federal University of Rio Grande do Norte, Natal, 59078-970 Brazil; 14 Laboratoire de Biosystématique et Ecologie des Arthropodes, Faculté des Sciences de la Nature et de la Vie, Université Frères Mentouri Constantine 1, 25000 Constantine, Algeria; 15 Section Ecology and Evolution, A-LIFE, Vrije Universiteit Amsterdam, De Boelelaan 1085, 1081 HV Amsterdam, The Netherlands; 16 Community and Conservation Ecology group, GELIFES, University of Groningen, PO Box 72, 9700 AB Groningen, The Netherlands; 17 Administración de Parques Nacionales, Calle Gral. San Martín y Padre Torrez (N3366), San Antonio, Misiones Argentina; 18 Systems Ecology, A-LIFE, Faculty of Science, Vrije Universiteit, 1081 HV Amsterdam, The Netherlands; 19 School of Biology and Environmental Science, University College Dublin, Belfield, Dublin, 4 Republic of Ireland; 20 Earth Institute, University College Dublin, Belfield, Dublin, 4 Ireland; 21 Department of wood and forest sciences, Université Laval, Québec, Qc G1V 0A6 Canada; 22 Instituto de Biologia de Solo, Universidade Estadual da Paraíba, Rua Horácio Trajano de Oliveira, 666, João Pessoa/PB, 58071-160 Brazil; 23 Department of Plant Protection, Bogor Agricultural University, Jalan Kamper, Kampus IPB Darmaga, 16680 Bogor, Indonesia; 24 Unidad Multidisciplinaria de Docencia e Investigación-Juriquilla, Facultad de Ciencias, Universidad Nacional Autónoma de México, Boulevard Juriquilla 3001, Juriquilla, Querétaro 76230 México; 25 Univ Rouen Normandie, INRAE, ECODIV USC 1499, F-76000 Rouen, France; 26 FiBL France, Research Institute of Organic Agriculture, pole bio - ecosite du val de Drome, 26400 Eurre, France; 27 Asian School of the Environment, Nanyang Technological University, 50 Nanyang Avenue, 639798 Singapore, Singapore; 28 Securing Antarctica’s Environmental Future, School of Biological Sciences, Monash University, Melbourne, Victoria 3800 Australia; 29 Ecology and Evolutionary Biology Department, University of Michigan, Ann Arbor, Michigan USA; 30 University of Michigan Biological Station, Pellston, Michigan USA; 31 CEFE, Université Paul-Valéry Montpellier 3, Université de Montpellier, CNRS, EPHE, IRD, route de Mende, 34000 Montpellier, France; 32 Institute of Soil Biology and Biogeochemistry, Biology Centre CAS, České Budějovice, Czech Republic; 33 Zoology, University of Autónoma de Madrid, C. Darwin, 2, 28049 Madrid, Spain; 34 Laboratório de Sistemática de Collembola e Conservação, Coleção de Referência de Fauna de Solo, Instituto de Biologia de Solo, Universidade Estadual da Paraíba, Campus V, Rua Horácio Trajano, 666 João Pessoa, Brazil; 35 UMR7205, Museum national d’Histoire naturelle, 45 rue Buffon, 75005 Paris, France; 36 CREAF, E08193 Bellaterra (Cerdanyola del Vallès), Catalonia, Spain; 37 Universitat Autònoma de Barcelona, E08193 Bellaterra (Cerdanyola del Vallès), Catalonia, Spain; 38 Department of Ecological Science, Vrije Universiteit Amsterdam, Amsterdam, the Netherlands; 39 Institute of Biology Bucharest, Romanian Academy, Bucharest, Romania; 40 University of Bremen, FB 02, UFT, General and Theoretical Ecology, Leobener Str. 6, D-28359 Bremen, Germany; 41 Department of Coastal Systems, Royal Netherlands Institute for Sea Research, ‘t Horntje, the Netherlands; 42 Insect Ecology Laboratory, Department of Forest Entomology, Forestry and Forest Products Research Institute, 1 Matsunosato, Tsukuba, Ibaraki, 305-8687 Japan; 43 Département des sciences biologiques, Université du Québec à Montréal, C.P. 8888 succ. Centre-ville, Montréal, Québec H3C 3P8 Canada; 44 Centre d’étude de la forêt -141, Avenue du Président-Kennedy, Montréal, Québec H2X 1Y4 Canada; 45 Department of Geography and Spatial Information Techniques, Ningbo University, 315211 Ningbo, China; 46 Zhejiang Collaborative Innovation Center & Ningbo Universities Collaborative Innovation Center for Land and Marine Spatial Utilization and Governance Research, Ningbo University, 315211 Ningbo, China; 47 Université du Québec en Outaouais, 58, rue Principale, Ripon, Qc J0V 1V0 Canada; 48 Laboratoire écologie fonctionnelle et environnement, Université de Toulouse, CNRS, Toulouse, 6 France; 49 Department of Plant and Soil Sciences, University of Pretoria, Private Bag X20, Hatfield, 0028 South Africa; 50 Department of Environmental System Science, Faculty of Science and Engineering, Doshisha University, 1-3 Tatara Miyakodani, Kyotanabe, Kyoto, 610-0394 Japan; 51 University of Applied Sciences and Arts of Western Switzerland, Geneva, 150 route de Presinge, 1254 Jussy, Switzerland; 52 Kyushu University Forest, Kyushu University, 394 Tsubakuro, Sasaguri, Fukuoka, 811-2415 Japan; 53 Department of Ecoscience, Aarhus University, C.F. Møllers Allé 4, 8000 Aarhus C, Denmark; 54 Departmento de Biogeoquímica, Ecología Vegetal y Microbiana/ Instituto de Recursos Naturales y Agrobiología de Sevilla (IRNAS), Consejo Superior de Investigaciones Científicas(CSIC), Avenida Reina Mercedes 10, 41012 Sevilla, Spain; 55 Tartu College, Tallinn University of Technology, Puiestee 78, 51008 Tartu, Estonia; 56 Institute of Agricultural and Environmental Sciences, Estonian University of Life Sciences, Kreutzwaldi Str. 5, Tartu, 51006 Estonia; 57 Department of Life Sciences, Aberystwyth University, Cledwyn Building, Penglais Campus, Aberystwyth, SY23 3DD Wales UK; 58 Department of Biological Sciences, University of Cape Town, Private Bag X3, Rondebosch, 7701 South Africa; 59 Research and Exhibitions Department, Iziko Museums of South Africa, 25 Queen Victoria Road, Cape Town, 8001 South Africa; 60 Université Paris-Saclay, INRAE, AgroParisTech, UMR EcoSys, 91120 Palaiseau, France; 61 Quantitative Ecology Lab, Department of Ecology, Universidade Federal do Rio Grande do Sul, Porto Alegre, RS 91540-000 Brazil; 62 Institute of Biology, University of Latvia, O.Vācieša Street 4, Riga, LV-1004 Latvia; 63 Agricultural University of Iceland, Hvanneyri, 311, Borgarbyggð, Iceland; 64 Department of Zoology, Institute of Biology and Ecology, Faculty of Science, Pavol Jozef Šafárik University, Košice, Slovakia; 65 Department of Soil and Environment, Swedish University or Agricultural Sciences, 750 07 Uppsala, Sweden; 66 Climate Impacts Research Centre, Umeå University, Abisko Scientitific Research Station, 98107 Abisko, Sweden; 67 Key Laboratory of the Three Gorges Reservoir Region’s Eco-Environment, Ministry of Education, Chongqing University, Chongqing, 400045 China; 68 Department of Biology, University of Western Ontario, 1151 Richmond Street, London, Ontario N6A 3K7 Canada; 69 Centre for Invasion Biology, Department of Botany and Zoology, Stellenbosch University, Private Bag X1, Matieland, 7602 South Africa; 70 Biología, Facultad de Ciencias, Universidad Autónoma de Madrid, Darwin 2. Cantoblanco, 28049 Madrid, España; 71 Institute of Zoology, Johannes Gutenberg University Mainz, 55128 Mainz, Germany; 72 Department of BioSciences, Rice University, Houston, TX 77005 USA; 73 Ecology & Zoology Group, School of Natural Sciences, Massey University, P.B, 11222 Palmerston North, New Zealand; 74 Department of Landscape Architecture, Gund Hall, 48 Quincy Street, Suite 312, Cambridge, MA 02138 USA; 75 Basque Centre for Climate Change – BC3, B/Sarriena s/n, 48940 Leioa, Spain; 76 Graduate School of Environment and Information Sciences, Yokohama National University, 79-7 Tokiwadai, Hodogaya, Yokohama, 240-8501 Japan; 77 Department of Sustainable Crop Production (DI.PRO.VE.S.), Università Cattolica del Sacro Cuore, Via Emilia Parmense 84, 29122 Piacenza, Italy; 78 Hawkesbury Institute for the Environment, Western Sydney University, Locked Bag 1797, Sydney, NSW 2751 Australia; 79 Department of Biology, IVAGRO, University of Cádiz, Campus de Excelencia Internacional Agroalimentario (CeiA3), Campus del Rio San Pedro, 11510 Puerto Real, Cádiz, Spain; 80 Laboratorio de Ecología, Dept. Ecología y Recursos Naturales, Facultad de Cienicas, UNAM, Ave. Universidad 3000, Copilco, Coyoacán 04510 CDMX Mexico; 81 Muséum National d’Histoire Naturelle, Department Adaptations du Vivant, UMR MECADEV, 4 avenue du Petit-Château, 91800 Brunoy, France; 82 Natural History Museum Vienna, 1. Zoology, Burgring 7, 1010 Vienna, Austria; 83 University of Natural Resources and Life Sciences, Department of Integrative Biology and Biodiversity Research, Institute of Zoology, Gregor-Mendel-Straße 33, 1180 Vienna, Austria; 84 Center of Excellence in Environmental Studies, King Abdulaziz University, P.O. Box 80216, Jeddah, 21589 Saudi Arabia; 85 Observatório Espeleológico, Avenida João Pinheiro, 607, Bairro Boa Viagem, Belo Horizonte, Minas Gerais CEP: 30.130-185 Brazil; 86 Department of Soil Zoology, Senckenberg Society for Nature Research, Görlitz, Germany; 87 Aix Marseille Univ, Avignon Univ, CNRS, IRD, IMBE, Marseille, France; 88 Forest Research Institute of the Karelian Research Centre of the Russian Academy of Sciences11 Pushkinskaya St, 185910 Petrozavodsk, Karelia Russia; 89 Lancaster Environment Centre, Lancaster University, Lancaster, LA1 4YQ UK; 90 Smithsonian Tropical Research Institute, Balboa, Ancón, Panama, Panama; 91 University of Natural Resources and Life Sciences Vienna, Department of Integrative Biology and Biodiversity Research, Institute of Zoology, Gregor-Mendel-Strasse 33, A-1180 Vienna, Austria; 92 Institute for Alpine Environment, Eurac Research, Drususallee 1, 39100 Bozen, Italy; 93 Universität Innsbruck, Department of Ecology, Technikerstrasse 25, 6020 Innsbruck, Austria; 94 School of Life and Health Sciences, Whitelands College, Holybourne Avenue, London, SW15 4JD UK; 95 Scientific department, State Nature Reserve “Privolzhskaya Lesostep”, Okruzhnaya, 12 a, 440031 Penza, Russia; 96 Institute of Systematics and Ecology of Animals of Siberian Branch of Russian Academy of Sciences (ISEA SB RAS), Moscow, Russia; 97 Museum and Institute of Zoology Polish Academy of Science, 00-679, Warsaw, Wilcza 64 Poland; 98 Key Laboratory of Urban Environment and Health, Ningbo Observation and Research Station, Institute of Urban Environment, Chinese Academy of Sciences, Xiamen, 361021 China; 99 Zhejiang Key Laboratory of Urban Environmental Processes and Pollution Control, CAS Haixi Industrial Technology Innovation Center in Beilun, Ningbo, 315830 China; 100 Institute of Biology of Komi Science Centre of the Ural Branch of the Russian Academy of Sciences, Moscow, Russia; 101 Tropical Ecology & Entomology Lab, Asian School of the Environment, Nanyang Technological University, Singapore. Address: 50 Nanyang Avenue, Singapore, 639798 Singapore; 102 Institute of Ecology and Evolution, University of Bern, Baltzerstrasse 6, 3012 Bern, Switzerland; 103 Data, Products and Society Node, South African Polar Research Infrastructure (SAPRI), 5th Floor, Foretrust Building, Martin Hammerschlag Way, Cape Town, 8000 South Africa; 104 Department of Ecology, School of Biology, Aristotle University of Thessaloniki, Biology Building, University Campus, P.O.119, 54124 Thessaloniki, Greece; 105 Natural Resources Canada, Canadian Forest Service, 1219 Queen St. E., Sault Ste, Marie, Ontario P6A 2E5 Canada; 106 Greensway AB, SE75651 Uppsala, Sweden; 107 Departement of Ecology, Swedish University of Agricultural Sciences, P.O. Box 7044, SE-75007 Uppsala, Sweden; 108 Department of Soil Science, IPB University, Jln. Meranti Kampus IPB Darmaga, Bogor, 16680 Indonesia; 109 Université Clermont Auvergne, INRAE, VetAgro Sup, UMR Ecosystème Prairial, 63000 Clermont-Ferrand, France; 110 Institute of Wildlife Biology and Management, University of Sopron, Bajcsy-Zs. str. 4, H-9400 Sopron, Hungary; 111 Key Laboratory of Wetland Ecology and Environment, Institute of Northeast Geography and Agroecology, Chinese Academy of Sciences, Changchun, 130102 China; 112 Key Laboratory of Vegetation Ecology, Ministry of Education, Northeast Normal University, Changchun, 130024 China; 113 State Environmental Protection Key Laboratory of Wetland Ecology and Vegetation Restoration, School of Environment, Northeast Normal University, Changchun, 130117 China; 114 Key Laboratory of Vegetation Ecology, Ministry of Education, Northeast Normal University, Changchun, Jilin 130024 China; 115 Community Department, Helmholtz Center for Environmental Research, Halle, Germany; 116 Department of Biology, Institute of Soil Biology, Paraiba State University campus V. Av. Horacio Trajano, #666, Cristo Redentor, 58070-450 João Pessoa, PB Brazil; 117 Key Laboratory for Earth Surface Processes of the Ministry of Education, Institute of Ecology, College of Urban and Environmental Science, Peking University, Beijing, China; 118 Ocean Program, World Resources Institute, London, UK; 119 Present Address: Department of Global Change Ecology, Biocenter, University of Würzburg, John-Skilton-Strasse 4a, 97074 Würzburg, Germany

**Keywords:** Biodiversity, Community ecology

## Abstract

Springtails (Collembola) inhabit soils from the Arctic to the Antarctic and comprise an estimated ~32% of all terrestrial arthropods on Earth. Here, we present a global, spatially-explicit database on springtail communities that includes 249,912 occurrences from 44,999 samples and 2,990 sites. These data are mainly raw sample-level records at the species level collected predominantly from private archives of the authors that were quality-controlled and taxonomically-standardised. Despite covering all continents, most of the sample-level data come from the European continent (82.5% of all samples) and represent four habitats: woodlands (57.4%), grasslands (14.0%), agrosystems (13.7%) and scrublands (9.0%). We included sampling by soil layers, and across seasons and years, representing temporal and spatial within-site variation in springtail communities. We also provided data use and sharing guidelines and R code to facilitate the use of the database by other researchers. This data paper describes a static version of the database at the publication date, but the database will be further expanded to include underrepresented regions and linked with trait data.

## Background & Summary

Soil biodiversity represents a major fraction of life on Earth^1,2^. Despite that, globally we know little about the current status and trends of soil life, especially invertebrates. Over the last few years, our knowledge on the global distribution of earthworms^3^, nematodes^4^, springtails^5^, ants^6^ and other macrofauna^7^ has advanced, showing trends different from aboveground biodiversity^8^. This urges us to deliver open and in-depth knowledge on soil animal life for nature conservation and for understanding the functioning of terrestrial ecosystems^9^. To help with this task, we here present a comprehensive fine-resolution database on the global distribution of springtails (Collembola), based on a compilation of published and unpublished data of researchers worldwide.

With literally worldwide distribution, springtails account for ~32% of the global terrestrial arthropod abundance^10^ and have global biomass of ~27.5 Megatonn carbon^5^. They are especially numerous in cold regions, but are also ubiquitous in tropical soils^5^, and even tropical canopies^11^. Springtails are central components of the belowground system, affecting litter decomposition, microbial activity, abundance and dispersal, and plant growth, and serving as food for numerous invertebrate predators^12^. Despite a moderate total diversity (~9500 described species^13^), springtail communities typically host dozens of species in a few square metres^5^. Due to their ubiquitous presence, and high abundance and local diversity, springtails represent an ideal model taxon for macroecological studies as well as bioindicators, but so far data limitations have constrained studies to address questions solely at local to regional scales.

In this paper, we describe a novel database mainly compiled from private archives of contributing authors that served as the basis for the recently published global synthesis study on springtail abundance and diversity^5^. While the site-level summaries of springtail community parameters have been published together with the synthesis^5^, here we present much more detailed sample-level data that include taxonomic names and 16 additional datasets (1398 new samples). With this effort, we complement the previously published data papers on nematodes^14^ and earthworms^15^ in describing the global soil invertebrate diversity. We also take a step further by providing quality-controlled species-level data with standardised taxonomic names at fine-scale resolution, i.e. from individual samples, or even soil layers, within each sampling site. Our dataset allows for both analyses of global and regional patterns of diversity and community composition, species distributions, and within-site variations in abundance and diversity. Below, we first describe how the data were collected, checked, curated, structured, and standardised, then we provide an overview of the data, and finish with some notes on how the data can be used.

## Methods

### Data sources

The database represents a standardised compilation of available datasets. The data were primarily obtained from individual archives of the contributing authors. To ensure widespread participation, the data collection initiative was announced openly in late summer 2019 through various channels, such as the mailing list of the International Colloquium of Apterygota and social media platforms such as Twitter and ResearchGate. Additionally, colleagues who had expertise in less well investigated regions, such as Africa and South America, were contacted through personal networks established by the initial author group. All individuals who collected, provided and standardised the data were invited to become co-authors of this study, with a defined minimum role in tasks, such as data provision, data cleaning, manuscript editing and approval. Both published^16–164^ and unpublished data were collected for analysis. Raw data, specifically species counts in samples, were requested whenever possible. Collection methods for the published data can be found in the original publications associated with each sampling event in the database. Furthermore, existing data on springtail communities available from Edaphobase^165^ were also included. To address the underrepresentation of Africa, South America, Australia, and Southeast Asia in the database, a literature search was conducted in January 2020 using the Web of Science platform with keywords: ‘springtail’ or ‘Collembola’ and ‘density’ or ‘abundance’ or ‘diversity’ along with the region of interest. In 2022–2023, in addition to the data analysed in the synthesis paper^5^, we included 16 datasets with 1,398 samples from new contributing authors.

The newly reported unpublished data represented 10,616 samples collected from 828 sites (from one to few dozens of samples were collected per site) and years 1975–2022. Springtails from soil and litter were collected using standard soil sampling devices (soil corers, frames). Collection from canopy was done using insecticide fogging, collections from aboveground surfaces were done using pitfall traps, stem eclectors, malaise traps, swipnetting, or vacuum cleaner. Over 90% of these data used different variations of Berlese or Kempson devices for springtail extraction. All springtails were identified under microscopes using regional identification keys (mainly to species, but also high-rank taxa or morphogroups). All sampling information for the entries in the dataset are included in the spreadsheet including the exact places, times, collectors, habitat types, and the collection and identification methods.

### Data collection

**Fig. 1:**
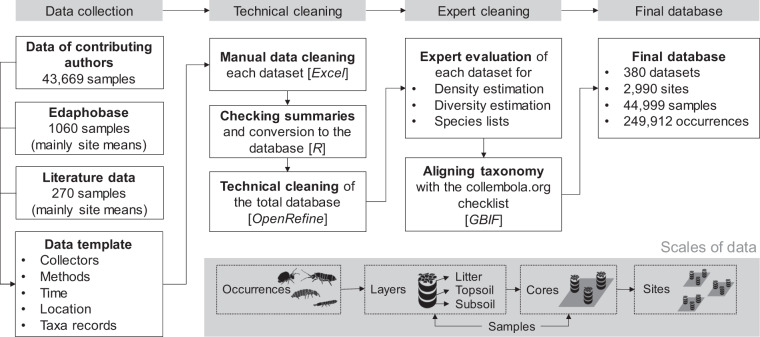
Data collection and evaluation in #GlobalCollembola. Most of the data are raw data collected from archives of the contributing authors of the paper. The data were collected using an Excel template and included in the final database after technical and expert cleaning of each dataset. No data were excluded, instead, expert evaluation is provided for each dataset. Whenever possible, we recorded species occurrences in individual samples (soil cores).

All data were entered into a common Microsoft Excel template (Supplementary materials Data template). The template included 30 columns describing the sampling approach and counts of springtail taxa. The following minimum set of variables was collected: collectors, collection method (including sampling area and depth), extraction method, identification precision and literature, collection date, latitude and longitude, and vegetation type (grassland, scrub, woodland, agriculture and other). Each contributed dataset was checked manually by a trained assistant for technical mistakes and completeness, and were complemented by authors if necessary. Geographical coordinates were checked using Google maps. We additionally performed descriptive statistics to check the consistency of the dataset (number of sites, samples, layers) and converted data in the template into two standard tables: events table (describing samples) and occurrence table (describing taxa counts) in R v. 4.0.2^166^ with RStudio interface v. 1.4.1103 (RStudio, PBC). The final events table across datasets was then checked for typos, consistency in vocabulary and outliers using OpenRefine v3.3 (https://openrefine.org; Fig. 1).

### Data evaluation

Every contributed dataset underwent a manual expert evaluation. Our evaluation process involved a board of springtail specialists, each with extensive research experience in specific geographic regions (expert names are listed in the events spreadsheet of the database). The experts individually scored each dataset based on three criteria: reliability of the (1) density, (2) species richness, and (3) the accuracy of the species names provided. The density estimation quality was determined by considering the sampling and extraction method, as well as the density estimation itself for the given ecosystem type. The species richness estimation quality and species names were assessed by considering the identification key used, the experience of the scientist identifying the animals, the species list and the species richness estimation itself for the given ecosystem type. Datasets that were deemed “unreliable” during the evaluation process were still included in the database, but the evaluation results by the experts are provided alongside the data.

### Taxonomic alignment

To make taxonomic lists comparable across contributed datasets, we checked all taxonomic names against the global checklist of Collembola (www.collembola.org). We did this using the ‘Species matching’ tool of the Global Biodiversity Information Facility (https://www.gbif.org/tools/species-lookup), which hosts the global checklist of Collembola from 2023. Original names were kept in the database together with the standardised names. For synonyms accepted species names were provided. For morphospecies described taxonomic names of higher ranks (usually genera) were given. Taxonomic hierarchy (genera, families, orders) and other taxonomic information was summarised in an additional spreadsheet. Unfortunately, it was not possible to fully control for factually wrong original identifications, even though the species lists were checked by experts (see above), but most of the records were judged as reliable.

## Data Records

**Fig. 2:**
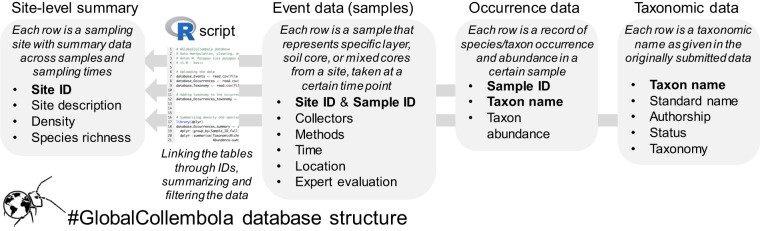
Database structure of #GlobalCollembola. Database consists of three main spreadsheets: (1) *Events*, (2) *Occurrences*, and (3) *Taxonomy*. The spreadsheets can be linked, summarised, and filtered using the associated R script to produce site-level averages.

The final dataset included 380 datasets representing 2,990 sites, 44,999 samples and 249,912 occurrences (i.e. observations of taxa in samples). In total, 1,441 taxa including 1,202 species were recorded in the occurrence data. The data were provided on different scales. Most samples represented single layers (i.e. litter, topsoil, deeper soil layers) in a soil core (i.e. soil monolith) or single cores in a sampling site (Fig. 1 ‘scales’). However, some data were available only as averages across samples at the sampling site level (typically an area up to a hundred of metres in diameter). The data were organised in three spreadsheets in the csv format: (1) *Events*, representing a list of all samples with described methodology, locations, and sampling times; (2) *Occurrences*, representing a list of all observations of taxa in all samples; and (3) *Taxonomy*, representing list of unique taxonomic names present in the occurrence data and associated standardised taxonomic names and other taxonomic information. Furthermore, we provided an R script to link the three spreadsheets together, summarise them by soil cores and sites, and filter unreliable data and data out of the scope (Fig. 2). As an example, we also provided a csv spreadsheet with average densities and the total species richness of springtails per site, collected with area-based methods. To facilitate data re-use, we provide a separate Excel spreadsheet with detailed descriptions of all data fields (‘Data description’). All data spreadsheets, R codes and other related information are available from Fighare^167^.

## Technical Validation

Statistical soundness of the database depends on the research question addressed. Below we show representativeness of our data for main types of ecological analysis by showing its spatial and temporal scopes, as well the sampling and identification approaches.

**Fig. 3:**
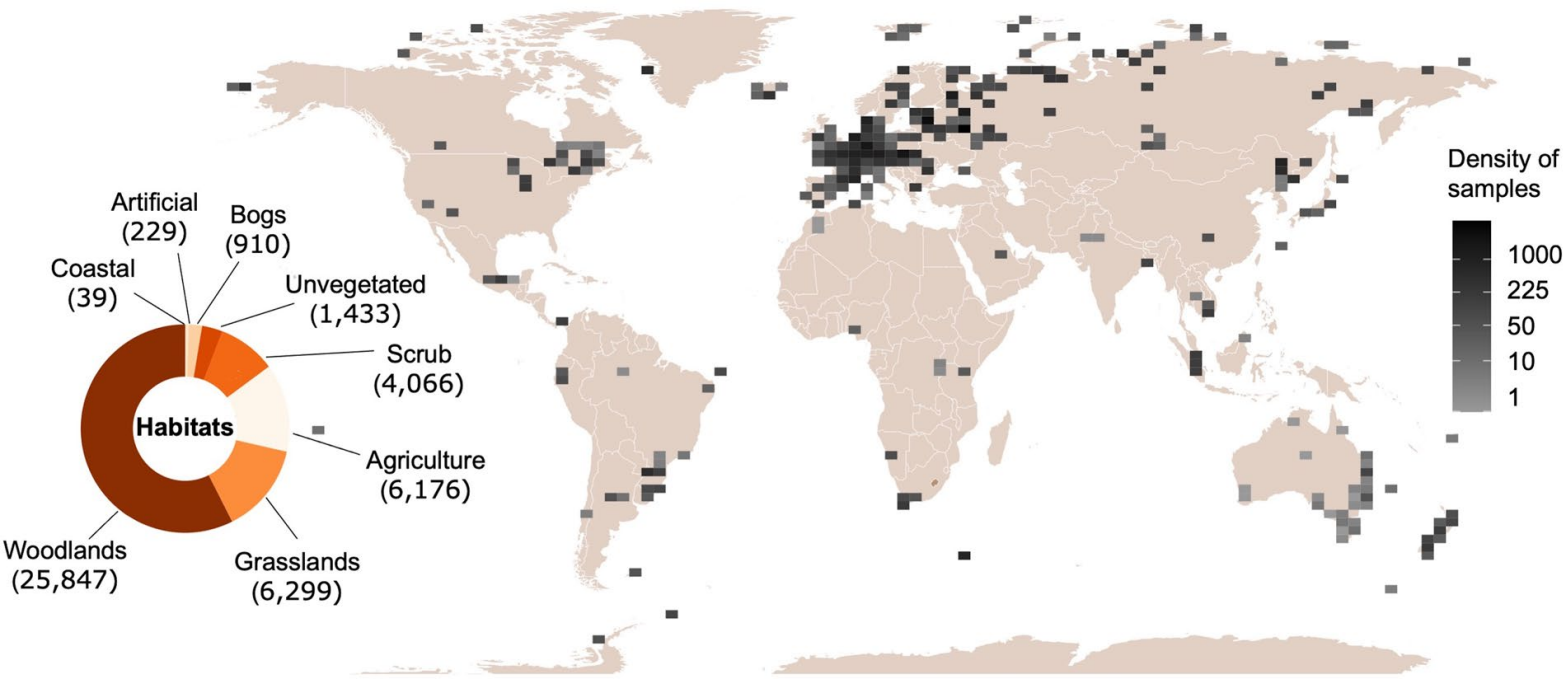
Global distribution of the sampling points and habitat types represented in the database. Density of samples per pixel in a global 100 × 100 coordinate grid are shown with grayscale (light – few samples, dark – many samples). Number of collected samples in each habitat type are shown with a doughnut chart; habitat classification follows the European Environmental Agency.

Most macroecological studies require representation of different geographical regions, climates, and ecosystem types^4^. Since the database is based on an open call for collection of already produced data, there is a clustered spatial distribution of data points in well-explored regions and high variation in collection methodologies. Most collected sample-level data come from Europe (82.5% or 37,137 samples), while other continents were less represented: Asia (5.6% or 2,508 samples), North America (3.4% or 1,528 samples), South America and the Caribbean (3.2% or 1,457 samples), Africa (2.8% or 1,269 samples), Australia (2.1% or 944 samples) and Antarctica (0.3% or 156 samples; Fig. 3). Across habitat types, woodlands are the most represented (57.4% of samples), followed by grasslands (14.0%), agriculture (13.7%), scrub (9.0%) and others (5.9%; Fig. 3). Using bootstrapping of the European data, we were able to do balanced analysis of the data in our synthesis study, and cover global gradients in mean annual temperature, precipitation, aridity, soil organic carbon content, pH, soil texture, vegetation biomass (NDVI), and habitat types (including the effects of agriculture)^5^. However, regional-scale analyses of the data are possible mainly in Europe, while tropical and subtropical regions, especially in Africa, are represented poorly.

**Fig. 4:**
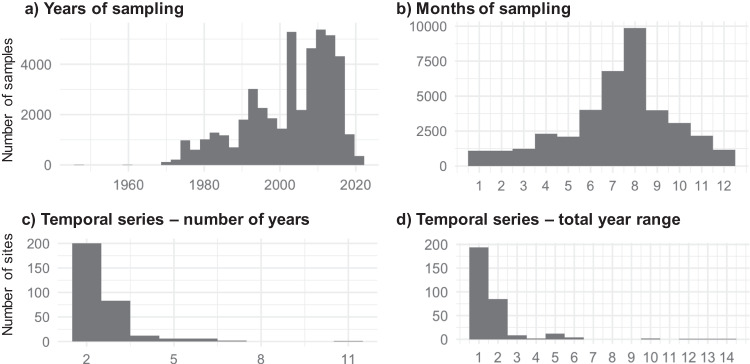
Temporal coverage of the database. Frequency histograms show the number of samples collected in different years (**a**) and months (**b**), and the number of sites where samples were collected in multiple years (**c**) in a certain time range (**d**).

Analyses of temporal variation, especially long-term changes of soil biodiversity^9^, require time series at different temporal scales. Seasonality is particularly important to consider when addressing macroecological questions, such as latitudinal biodiversity trends and their drivers^5^. Our database included records from years 1948–2022, with most data collected between 1975 and 2020 (Fig. 4a). Samples were collected throughout the year, with peak data collection in July-August (i.e., assumed peak springtail activity in northern Europe; Fig. 4b). There were 310 sites which were sampled in multiple years. Most of them were sampled only twice (Fig. 4c). However, 36 sites were sampled in 4 or more years and 5 sites were sampled over the range of 10 or more years (Fig. 4c,d). Therefore, it is possible to analyse long-term changes in springtail communities with two approaches: (1) by using available long-term monitoring data from few specific sites; (2) by using regional-scale data across different sites within specific habitat types sampled over decades (representative mainly for Europe, as the most studied region). It is also possible to account for seasonality in the global models because information on the sampling month is available for 86.4% of all sampling events^5^. However, the sampling is typically done in the periods of high springtail activities in each climate type^5^ and there is a clear data gap in the global temporal variation in springtail communities which should be addressed in the future data collections.

**Fig. 5:**
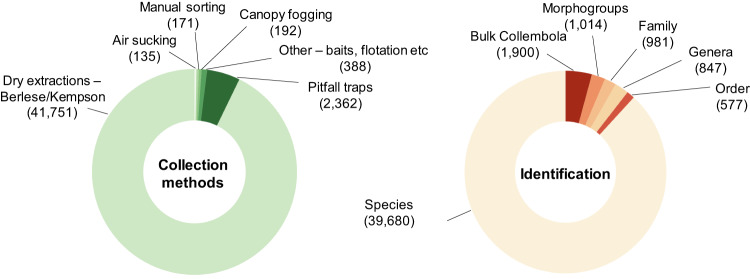
Collection methods and identification precision represented in the database. Number of collected samples with different methods and the number of samples where springtails were identified to a certain taxonomic resolution level are shown with doughnut charts.

Finally, comparability of different datasets in the database depends on the collection and identification methods. Records in the database represent mainly samples collected using area-based methods such as soil cores and animals extracted with heat (i.e. various modifications of Tullgren, Berlese, Kempson or Macfadyen extractors^168,169^; 92.8%, Fig. 5). Pitfall traps were the second most represented method (7.2%), and we included a single dataset collected using canopy fogging^11^. Most of the samples represented ‘soil’ (79.9% or 35,953 samples) and ‘litter’ microhabitats (54.8% or 24,676 samples). In total, 9,058 samples represented individual layers within soil cores, while 1,316 samples represented pooled data across samples within sampling sites. Therefore, data filtering and pooling is necessary to perform quantitative analyses of community metrics. In 88.2% of samples, springtails were identified to species level, while in 2.3% to morphogroups (typically roughly reflecting species-level diversity). For 4.2% of samples, springtails were recorded without further identification (abundance data only), while in the remaining records identification to order, families, or genera are provided (Fig. 5). Since most records in the database are species-level, the database is representative to evaluate global species-richness patterns and analyse species distributions in space and time.

## Usage Notes

Our global fine-resolution data on springtail communities can be openly used to address various (macro)ecological questions in space and time. Although our database is fully open, we encourage other researchers to follow our data usage and sharing guidelines: (1) the data can be openly used if a proper attribution to the data providers is given; (2) carefully evaluate representativeness of the data for your particular question; (3) report any issues you encounter; (4) we are there to support you – get in touch with the #GlobalCollembola expert community whenever you have questions. More detailed guidelines and the issue reporting form are available from Figshare together with the full database^167^.

For most research questions, different spreadsheets in the database need to be combined and summarised. We suggest that you use our R code for filtering and summarising the data. Please take special care while filtering the database – we kept unreliable records, and included data collected using different methods and with different sampling efforts. For analyses using species-level data, take care for synonymy of the taxa (see ‘canonical name’ and ‘species’ columns in the *Taxonomy* spreadsheet). As a note of caution, some species names represent complexes with cryptic genetic diversity^170,171^, or ambiguous (as in most invertebrate taxonomic systems), and thus interpretations about species distributions should be done with care.

The database, as a part of the #GlobalCollembola initiative, will be curated and continue to be expanded with contributions of new data. We also will upload our data to Edaphobase^165^ and GBIF^172^ for easier findability and better interoperability. This data paper describes a static version of the database at the publication date, while new updates will be available from other open online sources. We are also working on complementary trait and literature databases on springtails for community use, which will become openly available in upcoming years. This work is currently curated by the core committee of #GlobalCollembola, constituted of 20 volunteer data providers and experts.

Our database is useful for analyses of global and regional spatial patterns in springtail abundance and diversity^5^. The database includes time series data across seasons and years, and data on spatial variation within sites across samples and soil layers, allowing for in-depth analyses of dynamics of springtail communities. We also believe that the database is a valuable resource for species distribution modelling of soil organisms. All records in our database are the ‘event’ type of data, representing communities where all observed species are also recorded. This allows for reconstructing true absences by comparing species lists of different sites across datasets. Overall, we believe that our data will serve to answer multiple long-standing questions in soil ecology and conservation.

### Supplementary information

Template for data collection

Data Descriptor Worksheet

## Data Availability

Programming R code is openly available together with the database from Figshare^167^.
